# Comparative Microbiology and Antimicrobial Resistance of Bacterial Isolates from Wound Specimens at Tertiary-Care Centres in Ghana and Germany: A Retrospective Two-Centre Study

**DOI:** 10.3390/antibiotics15100959

**Published:** 2026-09-28

**Authors:** Edward Kwame Agyekum, Johanna Thiele, Mohammed Issah Suglo Bukari, Reginald Jeff Maddy, Jonas Roos, Ernst Molitor, Gunnar Thorben Rembert Hischebeth, Kristian Welle

**Affiliations:** 1 Department of Orthopedics and Trauma Surgery, Ho Teaching Hospital, Ho P.O. Box MA 374, Ghana; edkum130@gmail.com; 2 Department of Orthopedics and Trauma Surgery, University Hospital Bonn, 53127 Bonn, Germany; 3 Department of Orthopedics and Trauma Surgery, Tamale Teaching Hospital, Salaga/Yendi Road, Eastern Tamale, Tamale P.O. Box TL 16, Ghana; 4 Department of Orthopedics and Trauma Surgery, Cape Coast Teaching Hospital, Interberton Road, Cape Coast P.O. Box CT 1363, Ghana; ritteous@yahoo.com; 5 Institute of Medical Microbiology, Immunology and Parasitology, University Hospital Bonn, 53127 Bonn, Germany; molitor@uni-bonn.de (E.M.);

**Keywords:** antimicrobial resistance, wound infections, skin and soft tissue infections, Ghana, Germany

## Abstract

**Background**: Antimicrobial resistance complicates the management of skin and soft tissue infections (SSTIs), particularly in low- and middle-income countries where microbiological surveillance remains limited. We compared the microbiological spectrum and antimicrobial resistance patterns of wound-associated SSTIs between tertiary-care hospitals in Ghana and Germany. **Methods**: This retrospective bicentric cohort study analysed routine microbiological data from 309 patients in Ghana and 294 patients in Germany. 329 bacterial isolates from Ghana and 816 bacterial isolates from Germany were included. Pathogen distribution, antimicrobial susceptibility, and the prevalence of methicillin-resistant *Staphylococcus aureus* (MRSA), third-generation cephalosporin-resistant (3GC-R) *Enterobacterales*, and carbapenem-resistant Gram-negative bacteria were compared descriptively. **Results**: The microbiological spectrum differed markedly between the two cohorts. Gram-negative bacteria predominated in Ghana (83.9%), whereas Gram-positive organisms predominated in Germany (62.9%). *Escherichia coli*, *Pseudomonas aeruginosa*, and *Klebsiella* spp. were the leading pathogens in Ghana, while *Staphylococcus aureus* was the predominant pathogen in Germany. Compared with Germany, Ghanaian isolates exhibited substantially higher resistance to third-generation cephalosporins, fluoroquinolones and aminoglycosides. 3GC-R *Enterobacterales* were identified in 52.8% and 15.6% of evaluable isolates in Ghana and Germany, respectively. Carbapenem resistance among evaluable Gram-negative isolates was detected in 15.2% and 11.2%, respectively. MRSA prevalence (45.2% vs. 4.1%) was also considerably higher in Ghana than in Germany. **Conclusions**: Wound-associated SSTIs in Ghana and Germany differ substantially in pathogen distribution and antimicrobial resistance profiles. The predominance of Gram-negative pathogens and the high resistance burden observed in the Ghanaian cohort highlight the need for strengthened microbiological surveillance and antimicrobial stewardship. Institution-specific resistance data are essential to optimize empirical antimicrobial therapy across different healthcare settings.

## 1. Introduction

Antimicrobial resistance (AMR) is a major global public health threat, accounting for an estimated 1.2 million deaths directly and nearly five million deaths overall in 2019, with the highest burden in low- and middle-income countries (LMICs) [1]. The increasing prevalence of multidrug-resistant pathogens, including methicillin-resistant *Staphylococcus aureus* (MRSA), extended-spectrum β-lactamase (ESBL)-producing Enterobacterales and carbapenem-resistant Gram-negative bacteria, complicates the management of SSTIs by limiting empirical treatment options [2,3,4]. Therefore, continuous surveillance of local pathogen epidemiology and antimicrobial susceptibility patterns is essential for antimicrobial stewardship and evidence-based empirical therapy [5,6]. The epidemiology of skin and soft tissue infections (SSTIs) and antimicrobial resistance vary across regions and healthcare systems, reflecting differences in antimicrobial use, infection control, microbiological diagnostics, healthcare infrastructure, and stewardship programmes [3,4,5,6]. While high-income countries generally benefit from established surveillance systems and standardized diagnostics that support evidence-based treatment [5,7,8], many LMICs continue to face limited diagnostic capacity, insufficient surveillance, and inadequate antimicrobial stewardship, facilitating the emergence and spread of resistant pathogens [4,9].

Sub-Saharan Africa bears a disproportionate burden of infectious diseases and AMR [3,9]. In Ghana, wound infections are characterized by a predominance of Gram-negative pathogens and high resistance rates to commonly used antibiotics, including frequent ESBL-producing Enterobacterales and carbapenem-resistant *Acinetobacter baumannii* [10,11,12,13]. However, microbiological surveillance remains limited, with most data derived from single-centre studies [4,14]. In contrast, Germany benefits from established national and European AMR surveillance systems, although multidrug-resistant Gram-negative pathogens remain an important clinical challenge [15,16,17,18]. These surveillance programmes, however, may not adequately reflect pathogen distribution and susceptibility patterns in specific clinical settings such as wound-associated SSTIs [16,17]. Direct comparisons of wound microbiology and antimicrobial resistance between sub-Saharan Africa and Central Europe are scarce. Such analyses may improve understanding of regional differences in pathogen epidemiology and resistance patterns, thereby supporting empirical antimicrobial therapy and antimicrobial stewardship across diverse healthcare settings [3,4,5].

Wound-associated infections represent a particularly relevant setting for local antimicrobial resistance surveillance because their microbiological spectrum is heterogeneous and influenced by wound characteristics, chronicity, healthcare exposure, and previous antimicrobial therapy [19]. Empirical antimicrobial treatment may therefore vary considerably between institutions. While national or hospital-wide surveillance programmes provide important information on general resistance trends, institution- and unit-specific susceptibility patterns may differ substantially from aggregated data and may provide more relevant information for empirical antimicrobial treatment decisions [20,21,22]. Direct comparisons using harmonized analytical definitions may therefore provide clinically relevant information that cannot readily be derived from aggregate surveillance data alone. This retrospective study compared the microbiological spectrum and antimicrobial resistance patterns of bacterial isolates from wound-associated soft tissue infections at a teaching hospital in Ghana and a tertiary-care university hospital in Germany. We analysed pathogen distribution, antimicrobial susceptibility, and clinically relevant resistance phenotypes. By comparing two healthcare settings with distinct epidemiological backgrounds, this study aimed to improve understanding of centre-specific resistance patterns and to inform evidence-based empirical antimicrobial therapy.

The two centres were selected within an established clinical and scientific collaboration and provided contemporaneous microbiological datasets from tertiary-care settings with distinct healthcare and epidemiological backgrounds. Previous surveillance studies have demonstrated substantial setting-specific variation in pathogen distribution and antimicrobial resistance patterns, emphasizing the value of local microbiological surveillance [4,11]. The present study was therefore intended to compare institution-specific patterns rather than to provide nationally representative estimates for Ghana or Germany.

## 2. Materials and Methods

### 2.1. Study Design and Study Population

This retrospective, bicentric observational study compared the microbiological spectrum and antimicrobial resistance patterns of wound-associated soft tissue infections diagnosed between January and June 2026 at two tertiary-care hospitals located in distinct epidemiological settings: a university hospital in Bonn, Germany, and the Ho Teaching Hospital, a tertiary-care hospital in Ghana.

Routinely collected microbiological and demographic data were extracted from the respective institutional laboratory databases. Patients were eligible for inclusion if at least one wound-associated specimen had been submitted for bacterial culture as part of routine clinical care during the study period. Available demographic variables included age and sex. A patient was considered culture-positive when at least one wound specimen yielded one or more bacterial isolates. Only bacterial wound cultures obtained during routine clinical care were included.

Wound specimens were processed according to the routine diagnostic procedures of the respective microbiology laboratories. In the Ghanaian laboratory, bacterial identification was performed using the VITEK 2 system (bioMérieux, Marcy-l’Étoile, France), whereas antimicrobial susceptibility testing was performed using the Kirby–Bauer disc diffusion method. In the German laboratory, bacterial identification was performed by matrix-assisted laser desorption/ionization time-of-flight mass spectrometry (MALDI-TOF MS) using the VITEK MS PRIME system (bioMérieux). Antimicrobial susceptibility testing was performed using VITEK 2 systems (bioMérieux) and, where indicated, broth microdilution (UMIC, Bruker, Daltonics GmbH & Co. KG, Bremen, Germany), gradient diffusion methods (MIC Test Strips; Liofilchem, S.r.l., Roseto degli Abruzzi, Italy; ETEST, bioMérieux), and agar diffusion testing.

Because this retrospective study was based on routinely collected laboratory data, detailed clinical variables such as wound type and chronicity, previous antimicrobial exposure, comorbidities, and timing of specimen collection relative to antimicrobial treatment were not consistently available in a harmonized form across both cohorts. Consequently, these variables could not be included in adjusted analyses.

The study was approved by the local Institutional Review Board in Germany (approval no. 406/17) and in Ghana (approval no. HTH-REC/EX/2026/020).

### 2.2. Statistical Analysis

Categorical variables are presented as absolute numbers and percentages. Continuous variables are summarized as median and interquartile range (IQR). Analyses were descriptive and no adjustment for potential differences in case mix between the two cohorts was performed because harmonized individual-level clinical covariates were not available in both datasets. Pathogen distribution was analysed at the isolate level, whereas polymicrobial infections were assessed at the patient level. Antimicrobial resistance rates were calculated as the proportion of resistant isolates among all evaluable isolates for the respective antimicrobial agent or resistance phenotype and are reported together with the corresponding numerator and denominator. Organism–antibiotic combinations with fewer than 30 tested isolates were interpreted descriptively because of limited statistical precision.

### 2.3. Microbiological Analysis

Wound specimens were processed according to the routine diagnostic procedures of the respective microbiology laboratories. Antimicrobial susceptibility results were interpreted according to the European Committee on Antimicrobial Susceptibility Testing (EUCAST) criteria in both study cohorts.

Bacterial isolates were classified as Gram-positive or Gram-negative organisms. Prior to comparative analysis, bacterial nomenclature was harmonized between the two datasets by merging synonymous or overlapping microbiological designations. For selected species-specific analyses, closely related organisms were grouped at genus level, including *Klebsiella* spp., *Proteus* spp., *Citrobacter* spp., and *Acinetobacter* spp., to enable consistent comparison between both cohorts.

Pathogen distribution was analysed at the isolate level. For patient-level analyses, repeated recovery of the same bacterial species from specimens assigned to the same patient was counted only once. A polymicrobial infection was defined as the recovery of at least two different bacterial species from specimens assigned to the same patient. Polymicrobial infection rates were calculated using the number of culture-positive patients in each cohort as the denominator.

### 2.4. Antimicrobial Susceptibility Testing

Antimicrobial susceptibility testing (AST) results were interpreted according to EUCAST criteria in both study cohorts. Susceptibility categories were recorded as susceptible (S), susceptible with increased exposure (I), or resistant (R). Resistance rates were calculated using only isolates for which an interpretable AST result was available for the respective antimicrobial agent. Isolates without an available AST result for a given antimicrobial agent were excluded from the denominator for that specific analysis.

An isolate was considered evaluable for an antibiotic-specific resistance analysis when an S, I, or R result was available for the respective antimicrobial agent. For composite resistance phenotypes, an isolate was considered evaluable when the susceptibility information required to determine the respective phenotype was available. Accordingly, denominators varied between antimicrobial agents and resistance phenotypes and did not necessarily correspond to the total number of bacterial isolates in the respective cohort. Resistance was expressed as the proportion of resistant isolates among all evaluable isolates and is reported together with the corresponding numerator and denominator.

For the comparative antimicrobial resistance analysis, only antimicrobial agents with susceptibility results available in both study cohorts were included in the primary between-centre comparison. For Gram-negative organisms, these comprised amikacin (AM), gentamicin (GEN), amoxicillin–clavulanate (AMC), piperacillin–tazobactam (TZP), cefuroxime (CXM), cefotaxime (CTX), ceftriaxone (CRO), ceftazidime (CAZ), cefepime (CFP), meropenem (MEM), ciprofloxacin (CIP), levofloxacin (LFX), tetracycline (TE), and co-trimoxazole (SXT). For Gram-positive organisms, the comparative panel included penicillin (PEN), cefoxitin (FOX), erythromycin (E), azithromycin (AZ), clindamycin (CLI), gentamicin (GEN), ciprofloxacin (CIP), levofloxacin (LFX), tetracycline (TE), and co-trimoxazole (SXT). Antimicrobial agents tested exclusively in one cohort, including chloramphenicol in Ghana and imipenem (IPM), ertapenem (ERT), tobramycin (TOB), netilmicin (NET), doxycycline (DO), tigecycline (TIG), vancomycin (VA), teicoplanin (TEI), linezolid (LZD), tedizolid (TED), daptomycin (DAP), and fusidic acid (FA) in Germany, were excluded from the primary between-centre comparison. A complete list of antimicrobial agents and their abbreviations is provided in Supplementary Table S1.

### 2.5. Resistance Phenotypes

Methicillin-resistant *Staphylococcus aureus* (MRSA) was defined based on cefoxitin susceptibility testing in both study cohorts. In both laboratories, cefoxitin susceptibility was determined using the Kirby–Bauer disc diffusion method with a 30-µg cefoxitin disc and interpreted according to EUCAST criteria. In the German laboratory, the corresponding result was reported as oxacillin resistance in the routine antibiogram. Accordingly, *S. aureus* isolates documented as oxacillin resistant in the German dataset were classified as MRSA.

Third-generation cephalosporin-resistant *Enterobacterales* (3GC-R *Enterobacterales*) were defined as evaluable Enterobacterales classified as resistant to at least one tested third-generation cephalosporin (cefotaxime, ceftriaxone, and/or ceftazidime, depending on availability). This phenotype was used as a descriptive resistance endpoint and was not considered equivalent to confirmed extended-spectrum β-lactamase (ESBL) production, because confirmatory phenotypic or molecular ESBL testing was not consistently available. Other mechanisms of third-generation cephalosporin resistance, including AmpC β-lactamase production, could therefore not be excluded.

Carbapenem-resistant Gram-negative bacteria were defined as Gram-negative isolates classified as resistant to meropenem and/or imipenem, depending on the availability of susceptibility results. Only isolates with an available susceptibility result for at least one of these carbapenems were included in the denominator for this endpoint. Carbapenem resistance among *Acinetobacter* spp. was additionally assessed descriptively. Because the German cohort contained only one evaluable *Acinetobacter* isolate, no direct between-centre comparison of carbapenem resistance in *Acinetobacter* spp. was performed.

## 3. Results

### 3.1. Study Population and Microbiological Characteristics

The Ghanaian cohort comprised 309 patients (159 females, 150 males) with a median age of 42 years (IQR 24.0–58.5). Overall, 354 wound specimens yielded 329 bacterial isolates. In the German cohort, 294 patients (121 females, 173 males) with a median age of 59 years (IQR 44–73) contributed 499 wound specimens and 816 bacterial isolates after application of the predefined exclusion criteria. Polymicrobial infections were more frequent in Germany than in Ghana (52.9% [73/138] vs. 34.3% [85/248]). Gram-negative organisms predominated in Ghana (83.9%), whereas Gram-positive organisms predominated in Germany (62.9%). Demographic and microbiological characteristics are summarized in Table 1.

### 3.2. Pathogen Distribution

*Escherichia coli*, *Pseudomonas aeruginosa*, and *Klebsiella* spp. were the leading pathogens in Ghana, while *Staphylococcus aureus* and streptococci predominated in Germany. The comparative pathogen distribution is illustrated in Figure 1.

### 3.3. Antimicrobial Resistance

Overall resistance patterns differed between both cohorts (Figure 2). Ghanaian Gram-negative isolates showed markedly higher resistance to third-generation cephalosporins, fluoroquinolones and aminoglycosides than German isolates, whereas *Pseudomonas aeruginosa* remained comparatively susceptible to most antipseudomonal agents.

Among pooled Gram-negative isolates, resistance was generally higher in Ghana than in Germany, particularly to third-generation cephalosporins, fluoroquinolones and gentamicin, whereas resistance to piperacillin–tazobactam was slightly higher in Germany. Meropenem and amikacin resistance remained comparatively low in both cohorts (Figure 3A).

Among pooled Gram-positive isolates, resistance to penicillin and tetracycline was higher in Ghana, whereas higher resistance to clindamycin, gentamicin and fluoroquinolones was observed in Germany (Figure 3B). Several Ghanaian estimates were based on fewer than 30 tested isolates and should therefore be interpreted with caution. MRSA accounted for 45.2% (14/31) of *Staphylococcus aureus* isolates in Ghana compared with 4.1% (5/123) in Germany. Major resistance phenotypes are summarized in Table 2. 3GC-R *Enterobacterales* were identified in 52.8% of evaluable isolates in Ghana and 15.6% in Germany. Carbapenem-resistant Gram-negative bacteria were detected in 15.2% (27/178) of evaluable isolates in Ghana and 11.2% (30/267) in Germany. Among evaluable *Acinetobacter* spp. isolates in Ghana, 10/20 (50.0%) were carbapenem resistant. Because only one evaluable *Acinetobacter* isolate was available in the German cohort, no meaningful between-centre comparison was performed. (Table 2).

## 4. Discussion

The present bicentric study demonstrates substantial differences in the microbiological epidemiology and antimicrobial resistance patterns of wound-associated skin and soft tissue infections between a tertiary-care hospital in Ghana and a university hospital in Germany. While Gram-negative bacteria predominated among wound pathogens in Ghana, Gram-positive organisms, particularly *Staphylococcus aureus* and *streptococci*, were the leading pathogens in Germany. Moreover, isolates from Ghana exhibited considerably higher resistance to third-generation cephalosporins, fluoroquinolones and aminoglycosides, accompanied by a markedly higher prevalence of 3GC-R *Enterobacterales*, MRSA and carbapenem-resistant Gram-negative bacteria. These findings emphasize that the microbiology of wound infections differs substantially between healthcare settings and that empirical antimicrobial therapy should be guided by local surveillance data rather than extrapolated from international recommendations.

The predominance of Gram-negative pathogens in the Ghanaian cohort is consistent with previous studies from Ghana and other sub-Saharan African countries, which have repeatedly identified Enterobacterales and *Pseudomonas aeruginosa* among the leading pathogens isolated from chronic and postoperative wound infections [10,11,12,13]. In contrast, studies from Europe consistently report *Staphylococcus aureus* as the predominant pathogen in skin and soft tissue infections, followed by β-haemolytic streptococci, whereas Gram-negative organisms are generally restricted to chronic wounds, healthcare-associated infections or immunocompromised patients [2,15,17]. These differences probably reflect variations in healthcare infrastructure, wound chronicity, environmental exposure, infection prevention measures and referral patterns rather than geographical location alone [23]. Similar observations have been reported by the Global Burden of Bacterial Antimicrobial Resistance study, which demonstrated considerable regional heterogeneity in pathogen distribution and resistance profiles worldwide [3]. Beyond differences in pathogen distribution, the resistance patterns observed in this study have important clinical implications. More than half of evaluable Ghanaian Enterobacterales exhibited the 3GC-R phenotype, whereas approximately one sixth of evaluable isolates in Germany met the same definition. Likewise, resistance to cefotaxime, ceftriaxone and ciprofloxacin was markedly higher among Ghanaian *Escherichia coli* and *Klebsiella* spp. isolates. These findings are consistent with previous reports from Ghana, which describe widespread dissemination of ESBL-producing Enterobacterales in surgical-site infections and wound infections [10,11,12,13]. Similar trends have been observed throughout sub-Saharan Africa, where increasing resistance to third-generation cephalosporins has become a major challenge for empirical treatment of bacterial infections [4]. In contrast, the lower proportion of 3GC-R *Enterobacterales* observed in Germany is in accordance with data from the German Antibiotic Resistance Surveillance (ARS) programme and EARS-Net, where resistance remains comparatively low despite a gradual increase among Gram-negative pathogens in recent years [7,15,17]. Carbapenem resistance followed a similar pattern. In the Ghanaian cohort, 10 of 20 evaluable *Acinetobacter* spp. isolates were carbapenem resistant. However, only one evaluable *Acinetobacter* isolate was available in the German cohort, precluding a meaningful direct between-centre comparison. Carbapenem-resistant *Acinetobacter* has emerged as one of the most challenging healthcare-associated pathogens worldwide owing to its environmental persistence, ability to acquire resistance determinants, and potential to cause hospital outbreaks [24,25]. Although carbapenem resistance among Enterobacterales remained comparatively uncommon in both cohorts, the higher prevalence observed in Ghana is concerning because increasing carbapenem resistance threatens the effectiveness of last-line antimicrobial agents and underscores the importance of antimicrobial stewardship and infection prevention programmes [26,27].

The difference in MRSA prevalence between both centres further illustrates the distinct epidemiological settings. Approximately 45% of *Staphylococcus aureus* isolates in Ghana fulfilled the microbiological definition of MRSA, compared with only 4% in Germany. Cefoxitin-based phenotypic testing was used as the surrogate for methicillin resistance in both cohorts, allowing the MRSA classification to be harmonized between the two datasets [28,29]. Nevertheless, the marked difference in MRSA proportions should be interpreted in the context of potential differences in patient characteristics, healthcare exposure, antimicrobial use, and local epidemiology that could not be fully adjusted for in the present retrospective analysis. Similar regional variation has been described in previous African surveillance studies, where MRSA prevalence varies considerably between hospitals, emphasizing the importance of institution-specific surveillance rather than relying on national estimates alone [4].

One particularly interesting finding of the present study was the susceptibility profile of *Pseudomonas aeruginosa*. Despite representing one of the most frequently isolated Gram-negative pathogens in Ghana, resistance to ceftazidime, meropenem and ciprofloxacin remained unexpectedly low compared with Enterobacterales and *Acinetobacter* spp. This observation contrasts with the widespread assumption that all Gram-negative pathogens in high-resistance settings exhibit similarly elevated resistance rates. Instead, it demonstrates that antimicrobial resistance is highly species-specific and should not be generalized across bacterial groups [3,30]. Although the underlying reasons cannot be determined from the present retrospective dataset, several explanations appear plausible. In *Pseudomonas aeruginosa*, resistance may arise through multiple mechanisms, including chromosomal mutations, efflux-pump overexpression, porin alterations, inducible AmpC β-lactamase expression, and acquisition of transferable resistance determinants [30]. Consequently, local prescribing practices may influence resistance development differently among *Pseudomonas aeruginosa* than among *Enterobacterales*. If antipseudomonal β-lactams and carbapenems are reserved for severe infections or used more restrictively than broad-spectrum cephalosporins, selective pressure on *Pseudomonas aeruginosa* may remain comparatively limited. However, this explanation remains speculative because antimicrobial consumption data were not available. Alternative explanations include differences in local clonal epidemiology, environmental reservoirs or wound characteristics. The observed resistance patterns have direct implications for empirical antimicrobial therapy. In the Ghanaian cohort, the high prevalence of 3GC-R *Enterobacterales*, MRSA and carbapenem-resistant Gram-negative organisms suggests that empirical treatment regimens based on European resistance data may be insufficient. Conversely, the predominance of Gram-positive pathogens and comparatively lower resistance rates in Germany support narrower empirical antimicrobial coverage in appropriately selected patients. These findings underline the importance of institution-specific antibiograms and continuous microbiological surveillance as essential components of antimicrobial stewardship programmes [2,5].

The present study has several strengths. To our knowledge, it is among the first to directly compare the microbiological spectrum and antimicrobial resistance patterns of wound-associated skin and soft tissue infections between tertiary-care centres in sub-Saharan Africa and Central Europe using harmonized analytical methods. The study combined patient-, specimen- and isolate-level analyses, thereby providing a comprehensive characterization of pathogen epidemiology and resistance patterns. Furthermore, antimicrobial resistance was calculated using antibiotic-specific denominators, ensuring that resistance estimates were based exclusively on isolates with available susceptibility results. Finally, the comparison of institution-specific microbiological data from two distinct epidemiological settings provides clinically relevant information for empirical antimicrobial therapy and highlights the importance of local surveillance in guiding antimicrobial stewardship strategies.

Several limitations should be considered when interpreting the findings of this study. Importantly, the observed between-centre differences should not be interpreted as effects of geographical setting alone. The cohorts differed substantially in demographic characteristics, including age distribution, as well as in culture positivity. Furthermore, harmonized data on wound type and chronicity, previous antimicrobial exposure, comorbidities, referral patterns, and timing of specimen collection relative to antimicrobial treatment were not consistently available. These factors may independently influence both pathogen recovery and antimicrobial resistance patterns. Because corresponding individual-level covariates were unavailable in both datasets, adjusted analyses could not be performed. The findings should therefore be interpreted as descriptive, centre-specific comparisons rather than as causal effects of geographical location. Second, microbiological identification and antimicrobial susceptibility testing were performed according to local laboratory procedures, which may have introduced minor methodological differences despite harmonized resistance definitions. Third-generation cephalosporin resistance among Enterobacterales was used as a descriptive resistance endpoint. Confirmatory phenotypic or molecular testing for specific β-lactamase mechanisms was not consistently available; therefore, ESBL production, AmpC β-lactamase production, and other resistance mechanisms could not be reliably distinguished. Finally, the study was conducted at two tertiary-care centres and therefore may not be representative of the overall epidemiology of wound-associated infections in Ghana or Germany. Nevertheless, the findings provide valuable comparative data from two distinct epidemiological settings and highlight the importance of institution-specific microbiological surveillance.

Future studies should prospectively investigate wound-associated infections using harmonized clinical and microbiological definitions across different healthcare settings. In particular, standardized information on wound type and chronicity, previous antimicrobial exposure, comorbidities, healthcare exposure, timing of specimen collection, and clinical outcomes should be incorporated to allow adjustment for differences in case mix. Standardized susceptibility testing and, where feasible, phenotypic or molecular characterization of relevant resistance mechanisms would further help to distinguish differences attributable to patient characteristics from those related to local pathogen epidemiology. Multicentre studies involving several institutions within each country are also needed to determine whether the centre-specific patterns observed in the present study are representative of broader regional trends.

In conclusion, this retrospective two-centre study identified substantial differences in the microbiological spectrum and antimicrobial resistance patterns of wound-associated infections between tertiary-care centres in Ghana and Germany. Gram-negative organisms predominated in the Ghanaian cohort and showed comparatively high resistance to several commonly used antimicrobial agents, whereas Gram-positive organisms predominated in the German cohort. These findings emphasize the importance of institution-specific microbiological surveillance and locally adapted empirical treatment strategies. However, differences in patient characteristics, clinical case mix, previous antimicrobial exposure, and local diagnostic practices may have contributed to the observed patterns and preclude attribution of these differences to geographical setting alone. Prospective multicentre studies using harmonized clinical and microbiological protocols are required to further investigate these observations and their implications for antimicrobial stewardship.

## Figures and Tables

**Figure 1:**
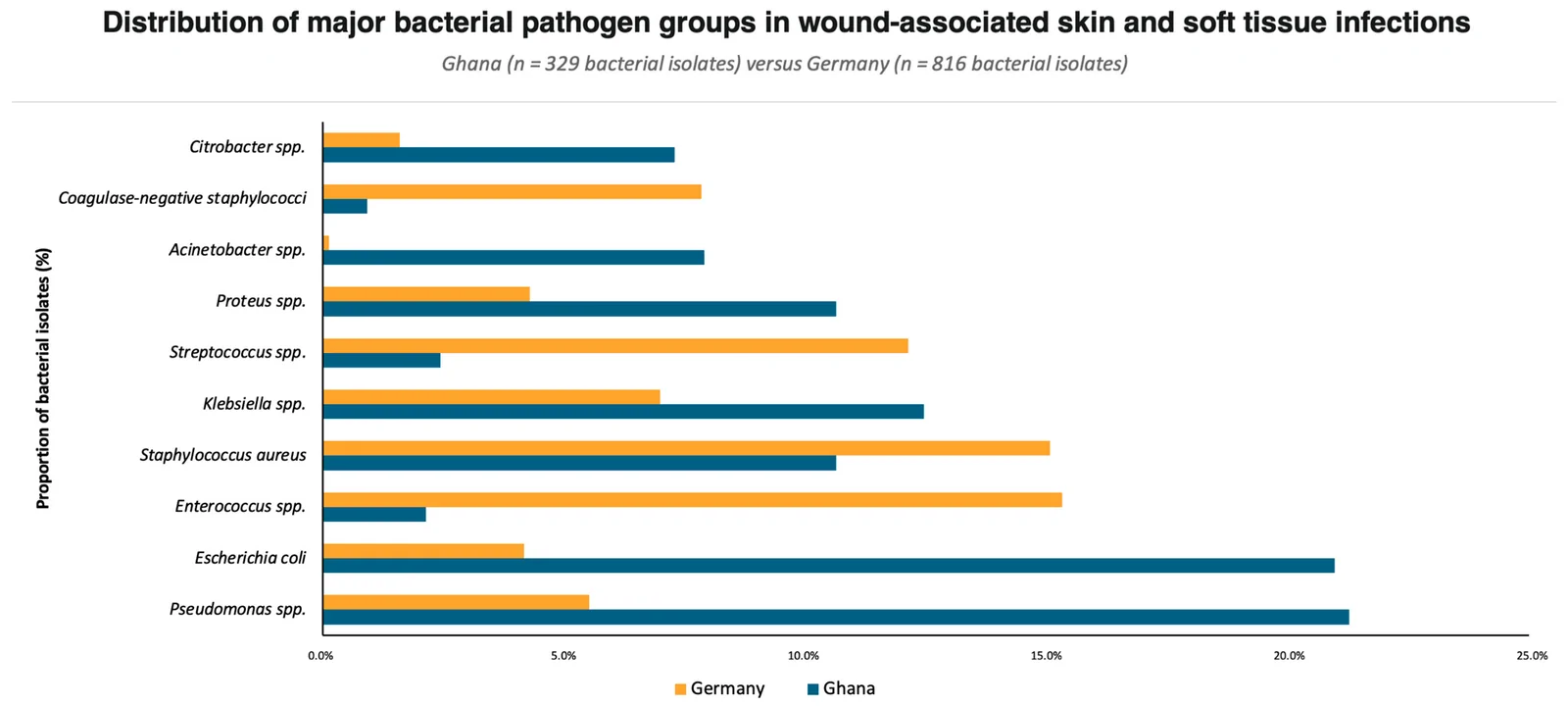
Relative distribution of the most frequently isolated bacterial pathogen groups from wound-associated skin and soft tissue infections in the Ghanaian and German cohorts. The x-axis represents the relative proportion (%) of each pathogen group among all bacterial isolates within the respective cohort, whereas the y-axis lists the bacterial species or genus groups included in the comparison. Percentages were calculated separately for Ghana and Germany using the total number of bacterial isolates in each cohort as the denominator.

**Figure 2:**
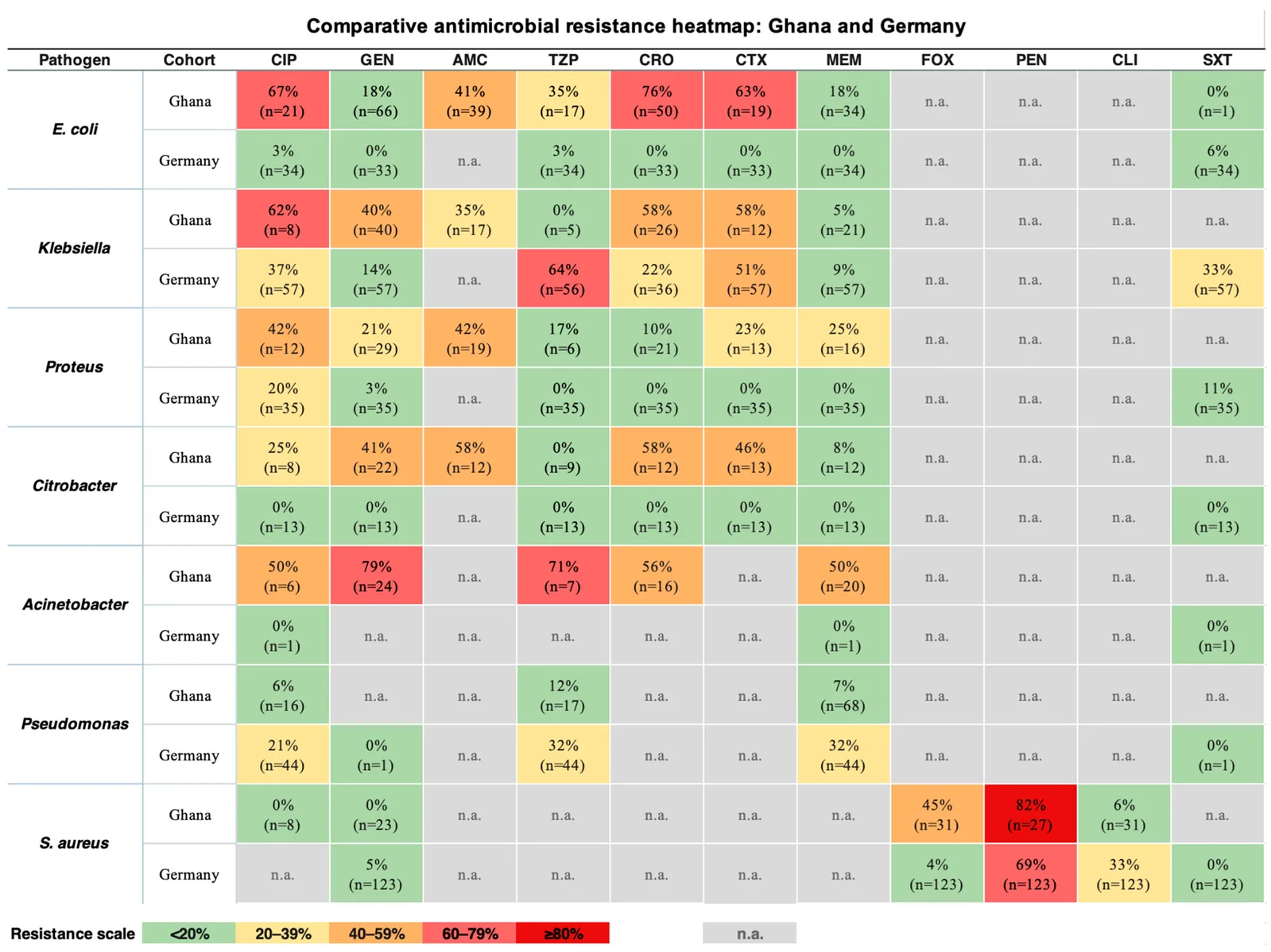
Comparative antimicrobial resistance heatmap for selected bacterial pathogens isolated from wound-associated skin and soft tissue infections in the Ghanaian and German cohorts. Cell labels indicate the percentage of resistant isolates among all evaluable isolates tested for the respective pathogen–antimicrobial combination and the corresponding number of tested isolates (*n*). Isolates without an available susceptibility result were excluded from the denominator. Grey cells (n.a.) indicate unavailable or non-comparable susceptibility results. Estimates based on fewer than 30 tested isolates should be interpreted with caution. Cell colours indicate increasing resistance categories from green to red (<20%, 20–39%, 40–59%, 60–79%, and ≥80%). CIP, ciprofloxacin; GEN, gentamicin; AMC, amoxicillin–clavulanate; TZP, piperacillin–tazobactam; CRO, ceftriaxone; CTX, cefotaxime; MEM, meropenem; FOX, cefoxitin; PEN, penicillin; CLI, clindamycin; SXT, co-trimoxazole.

**Figure 3:**
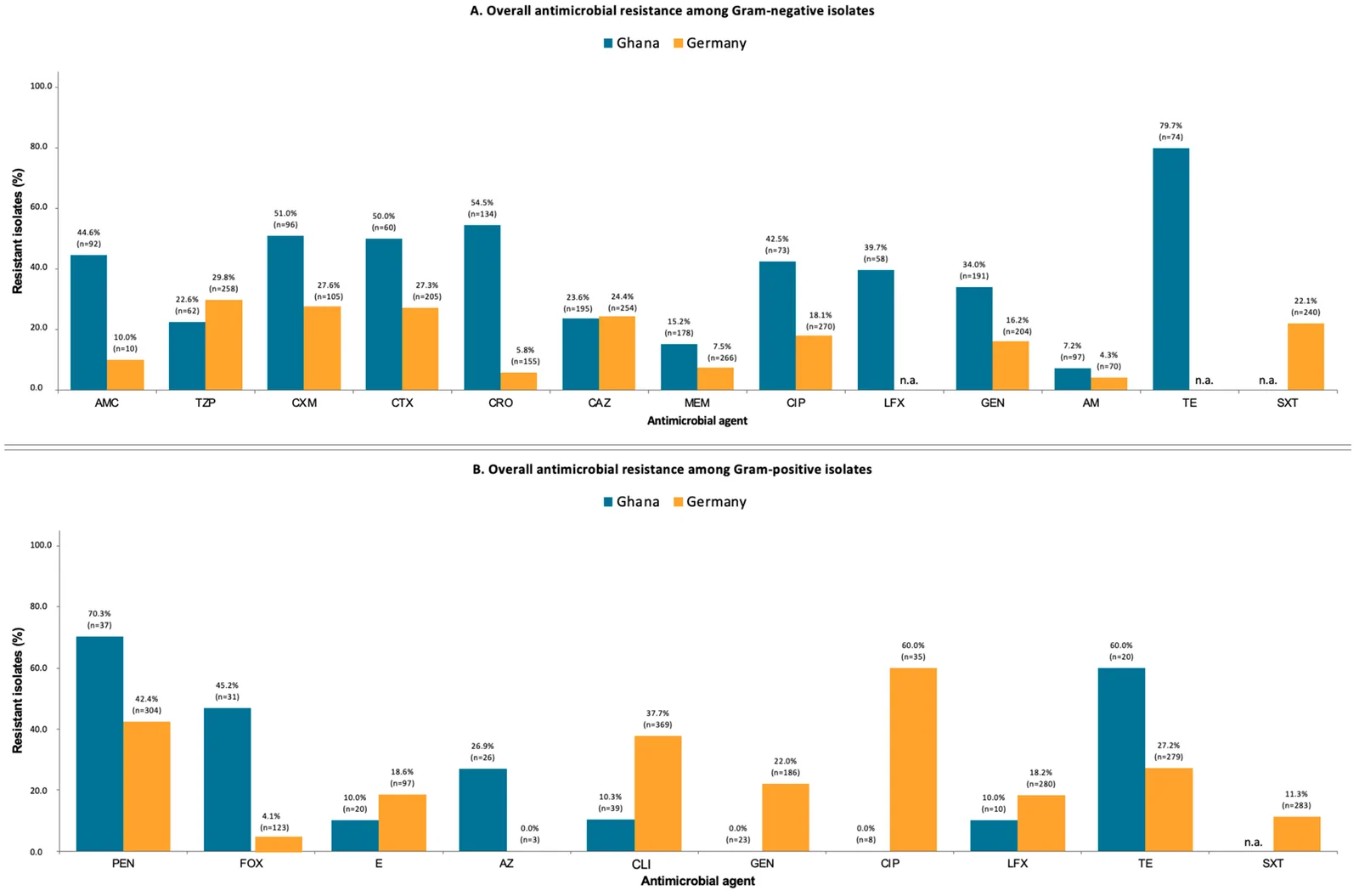
Overall antimicrobial resistance among Gram-negative and Gram-positive bacterial isolates in the Ghanaian and German cohorts. (**A**) shows resistance among Gram-negative isolates, and (**B**) shows resistance among Gram-positive isolates. Bars represent the percentage of resistant isolates among all evaluable isolates with an available susceptibility result for the respective antimicrobial agent. Values displayed for each bar indicate the resistance percentage and the corresponding number of tested isolates (*n*). Isolates without an available susceptibility result for the respective antimicrobial agent were excluded from the denominator. FOX values refer to *S. aureus* isolates evaluated for methicillin resistance. Entries marked “n.a.” indicate that susceptibility data were not available or were not suitable for between-centre comparison. AMC, amoxicillin–clavulanate; TZP, piperacillin–tazobactam; CXM, cefuroxime; CTX, cefotaxime; CRO, ceftriaxone; CAZ, ceftazidime; MEM, meropenem; CIP, ciprofloxacin; LFX, levofloxacin; GEN, gentamicin; AM, amikacin; TE, tetracycline; SXT, co-trimoxazole; PEN, penicillin; FOX, cefoxitin; E, erythromycin; AZ, azithromycin; CLI, clindamycin. A complete list of antimicrobial agents and abbreviations is provided in Supplementary Table S1.

**Table 1:** Demographic and microbiological characteristics of the study populations.

Variable	Ghana	Germany
Patients, n	309	294
Female, n (%)	159 (51.5)	121 (41.3)
Male, n (%)	150 (48.5)	173 (58.7)
Median age, years (IQR)	42 (24.0–58.5)	59 (44–73)
Wound specimens, n	354	499
Culture-positive patients, n (%)	248 (80.3)	138 (46.9)
Bacterial isolates, n	329	816
Gram-positive isolates, n (%)	53 (16.1)	513 (62.9)
Gram-negative isolates, n (%)	276 (83.9)	303 (37.1)
Polymicrobial infections, n (%)	85/248 (34.3)	73/138 (52.9)

**Table 2:** Comparison of major antimicrobial resistance phenotypes.

Resistance Phenotype	Ghana	Germany
MRSA	14/31 (45.2%)	5/123 (4.1%)
3GC-R Enterobacterales	94/178 (52.8%)	20/128 (15.6%)
Carbapenem-resistant Gram-negative bacteria	27/178 (15.2%)	30/267 (11.2%)
Ciprofloxacin-resistant Gram-negative bacteria	31/73 (42.5%)	49/270 (18.1%)
Gentamicin-resistant Gram-negative bacteria	65/191 (34.0%)	33/204 (16.2%)

## Data Availability

The datasets generated and analysed during the current study are not publicly available due to ethical and institutional data-protection requirements, as they contain pseudonymized patient-level clinical and microbiological information. De-identified data supporting the findings of this study may be made available by the corresponding author upon reasonable request, subject to approval by the participating institutions and in accordance with applicable data-protection regulations.

## Supplementary Materials

The following supporting information can be downloaded at https://www.mdpi.com/article/10.3390/antibiotics15100959/s1, Table S1: Abbreviations of antimicrobial agents included in the comparative antimicrobial resistance heatmap of bacterial pathogens isolated from wound-associated skin and soft tissue infections in Ghana and Germany.
