# Supplementary material for: Comparative Microbiology and Antimicrobial Resistance of Bacterial Isolates from Wound Specimens at Tertiary-Care Centres in Ghana and Germany: A Retrospective Two-Centre Study
Source: Antibiotics (Basel). 2026 Sep 28;15(10):959. doi: 10.3390/antibiotics15100959 (PMC13700649; doi:10.3390/antibiotics15100959)
Supplement: Supplementary file 1 [file antibiotics-15-00959-s001.zip › antibiotics-4505892-supplementary.pdf]

Supplemental

Table S1: Abbreviations of antimicrobial agents included in the comparative antimicrobial resistance heatmap of bacterial pathogens isolated from wound-associated skin and soft tissue infections in Ghana and Germany.

Abbreviation	Antimicrobial agent	Antimicrobial class
AM	Amikacin	Aminoglycoside
AMC	Amoxicillin/clavulanic acid	Aminopenicillin + β-lactamase inhibitor
AZ	Azithromycin	Macrolide
CAZ	Ceftazidime	Third-generation cephalosporin
CFP	Cefepime	Fourth-generation cephalosporin
CIP	Ciprofloxacin	Fluoroquinolone
CLI	Clindamycin	Lincosamide
CRO	Ceftriaxone	Third-generation cephalosporin
CTX	Cefotaxime	Third-generation cephalosporin
CXM	Cefuroxime	Second-generation cephalosporin
DAP	Daptomycin	Lipopeptide
DO	Doxycycline	Tetracycline
E	Erythromycin	Macrolide
ERT	Ertapenem	Carbapenem
FA	Fusidic acid	Steroid antibacterial
FOX	Cefoxitin	Cephamycin
GEN	Gentamicin	Aminoglycoside
IPM	Imipenem	Carbapenem
LFX	Levofloxacin	Fluoroquinolone
LZD	Linezolid	Oxazolidinone
MEM	Meropenem	Carbapenem
NET	Netilmicin	Aminoglycoside
PEN	Penicillin G	Natural penicillin
SXT	Trimethoprim/sulfamethoxazole	Folate pathway inhibitor
TE	Tetracycline	Tetracycline
TED	Tedizolid	Oxazolidinone
TEI	Teicoplanin	Glycopeptide
TIG	Tigecycline	Glycylcycline
TOB	Tobramycin	Aminoglycoside
TZP	Piperacillin/tazobactam	Ureidopenicillin + β-lactamase inhibitor
VA	Vancomycin	Glycopeptide
